# Effect of Infill Density in FDM 3D Printing on Low-Cycle Stress of Bamboo-Filled PLA-Based Material

**DOI:** 10.3390/polym14224930

**Published:** 2022-11-15

**Authors:** Miroslav Müller, Petr Jirků, Vladimír Šleger, Rajesh Kumar Mishra, Monika Hromasová, Jan Novotný

**Affiliations:** 1 Department of Material Science and Manufacturing Technology, Faculty of Engineering, Czech University of Life Sciences Prague, Kamycka 129, 165 00 Prague, Czech Republic; 2 Department of Mechanical Engineering, Faculty of Engineering, Czech University of Life Sciences Prague, Kamycka 129, 165 00 Prague, Czech Republic; 3 Department of Electrical Engineering and Automation, Faculty of Engineering, Czech University of Life Sciences Prague, Kamycka 129, 165 00 Prague, Czech Republic; 4 Faculty of Mechanical Engineering, J. E. Purkyne University in Usti nad Labem, Pasteurova 3334/7, 400 01 Usti nad Labem, Czech Republic

**Keywords:** 3D printing, PLA polymer, bamboo, low-cycle test, infill density, mechanical properties, SEM

## Abstract

In this paper, the fatigue behavior of polylactic acid (PLA) material with bamboo filler printed by 3D additive printing using fused deposition modelling (FDM) technology at different infill densities and print nozzle diameters is investigated. The mechanical test results are supported by the findings from SEM image analysis. The fatigue behavior was tested at four consecutive 250 cycles at loads ranging from 5 to 20, 30, 40, and 50% based on the limits found in the static tensile test. The results of the static tensile and low-cycle fatigue tests confirmed significant effects of infill density of 60%, 80%, and 100% on the tensile strength of the tested specimens. In particular, the research results show a significant effect of infill density on the fatigue properties of the tested materials. The influence of cyclic tests resulted in the strengthening of the tested material, and at the same time, its viscoelastic behavior was manifested. SEM analysis of the fracture surface confirmed a good interaction between the PLA matrix and the bamboo-based filler using nozzle diameters of 0.4 and 0.6 mm and infill densities of 60%, 80%, and 100%. Low-cycle testing showed no reductions in the mechanical properties and fatigue lives of the 3D printed samples.

## 1. Introduction

The current trend in the field of polymeric materials and related composites is towards the use of biological material, not only in the field of processing by injection moulding, vacuum infusion, and bonding technology, but also in the field of 3D printing. The use of biological material in the field of vacuum infusion and bonding technology is mainly related to the filler and the matrix, which are usually made of a synthetic resin. In the field of polymer injection moulding and 3D printing, both the biological matrix and the filler are used [1].

New materials, known as biocomposites, are emerging and belong to a group of materials with significant research potential. Among them, PLA is one of the most widely used biodegradable filament materials in the field of additive technologies [2]. However, its disadvantages include brittleness, low toughness, and flexibility [3]. Biocomposite filaments include materials with biodegradable polymeric matrices and bio-based fillers. The fillers can be in the form of particles or fibres. Particulate fillers are mainly used for additive technologies due to the nozzle deposition of the material. The filler content is usually up to 50% by volume when using natural materials [4]. The literature suggests a wide variation in the natural fillers added to the printing material used in 3D printing, especially for sustainability reasons [5,6,7]. The most widely used thermoplastic polymer in biocomposites in 3D printing is PLA. Fibre manufacturers use many types of cellulose or other natural fibres or particles as filler. The fibres used include: bamboo, birch, cherry, cedar, coconut, cork, ebony, olive, pine, or willow [8]. Research results show that bio-based fillers in combination with additive technology reduce production costs [9,10]. Examples are discoveries reported on kenaf particles, wood flour, etc. [9,10] However, changes in the mechanical properties of the filament must also be considered, which are highly dependent on the bio-based filler used, its treatment, and the interaction between the filler and the matrix [7,11,12,13,14,15]. The natural filler provides considerable variability in the microstructures and compositions of the different species [12].

There are many different technologies in the field of additive manufacturing or 3D printing. The most common and very popular method among users is additive FDM (fused deposition modelling) technology, which works with printing filament. This filament can be based on primary, secondary, or composite materials using different fillers [1,6]. The components printed using additive FDM technology typically have poorer mechanical behaviours than the same materials processed by injection moulding, stamping, or extrusion [16,17]. The quality of 3D-printed components, and consequently, their mechanical properties, are influenced by various parameters affecting interlayer fusion, porosity, and swelling caused by natural fibres and particles, etc. [17,18] On the other hand, additive technology offers designers more freedom and the possibility of addressing specific problems on an object, for example, by increasing the infill density [16,17]. Additive manufacturing involves the production of material in layers and is highly dependent on the printing input parameters [19]. The mechanical behaviour of 3D-printed materials is influenced by the diameter of the nozzle used, the material and any filler, the orientation of the layers and their thicknesses, the feed rate of the nozzle, the angle and width of the grid, and, last but not least, the density of the filler [20,21,22,23,24,25]. It is important to describe the mechanical properties not only by static tests but also by tests simulating cyclic loading, which are more relevant for practical applications. This is due to the deformation and possible destruction of the materials subjected to cyclic loading, which is undesirable. If the cyclic stress exceeds the elastic limit, plastic deformation accumulates, which then causes the material to gradually fail [10,18,25,26,27,28,29,30].

In this article, the suitability of bamboo-filled PLA filament for 3D printing applications is investigated with emphasis on fatigue behaviour. The mechanical properties are investigated based on the tensile test results and fatigue behaviours in cyclic tensile tests on printed test samples using FDM 3D printing technology of bamboo-filled PLA material. The aim of the research is to determine the basic mechanical characteristics and fatigue behaviours under different intensities of progressive cyclic loading for tensile tests for samples with different infill densities and nozzle diameters of 0.4 mm and 0.6 mm. The effects were studied on 3D printing parameters, including a confirmation whether the incorporation of a bamboo-based filler into PLA provides the expected mechanical benefits from a functional point of view, i.e., fatigue loading. The use of bio-based filler added to the filament is substantial, especially in terms of aesthetic significance visible on the surface [31].

## 2. Materials and Methods

### 2.1. Additive Production of Test Samples

The filament used for the research was a PLA-based filament from (AzureFilm, Sežana, Slovenia) and a bamboo filler. The material composition was 40% bamboo particles and 60% PLA polymer. The particle size was a trade secret of the company supplying this filament. The approximate size of the natural filler was determined by optical analysis using (Gwyddion, Jihlava, Czech Republic) and scanning electron microscopy (SEM) images. The approximate size of the natural bamboo filler in the PLA matrix was 22.2 ± 8.7 µm. SEM images before loading were evaluated.

**Figure 1:**
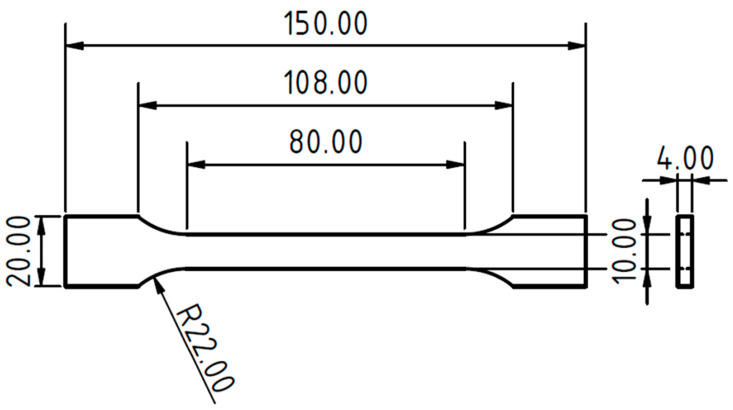
Test sample type 1B according to the ČSN EN ISO 527-2 created by 3D printing from PLA-based filament and bamboo filler.

A 3D printer using the Prusa i3 MK3S FDM technology (Prusa Research, a.s., Prague, Czech Republic) was used to produce the type 1B tensile test samples in accordance with ČSN EN ISO 527-2 (Figure 1).

**Figure 2:**
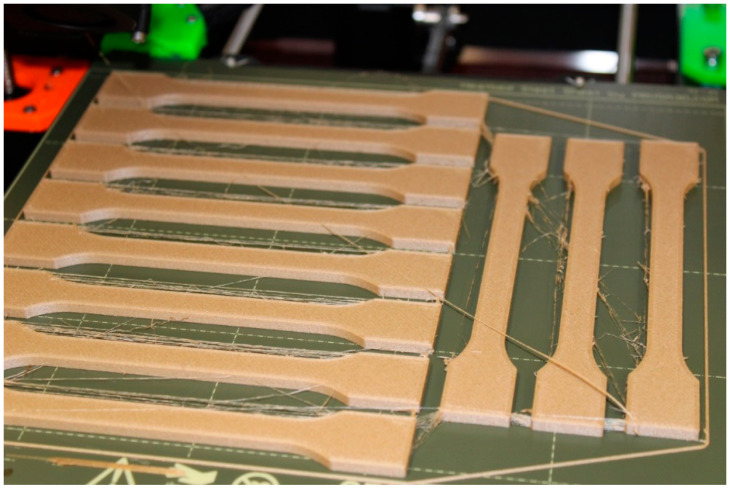
3D printing kit for PLA/B/nozzle 0.4 mm/60% test samples.

The 3D printer used supports a filament with a diameter of 1.75 mm, so this variant with the mentioned diameter and a spool weight of 750 g was chosen. The print files of the test samples were created in the open-source program (PrusaSlicer 2.5.0, Prague, Czech Republic). Two types of nozzles with diameters of 0.4 mm and 0.6 mm were selected for printing the samples. The nozzle diameter mainly affected the resulting mechanical properties of the 3D-printed sample and the overall printing time. Six basic series of samples were selected, as given in Table 1. The layer height was chosen to be 0.2 mm for all samples. Another common element was the type of filler chosen. It was a straight filler applied at an angle of 45° to the *X*-axis. The printing temperature was chosen to be 215 °C with a heated pad temperature of 60 °C. Printing was carried out in a closed environment of the print chamber, which reduces the risk of external influences affecting the print. As a result, the internal temperature in the print chamber was in the range of 22–26 °C during printing. To compare the weights, a series of pure PLA filament samples with a straight fill density of 100% was created with the same printing parameters as the previous samples. The printing of the test samples was relatively easy and smooth, and despite initial concerns, there was no jamming of the composite filament in the nozzle. Occasional stringing occurred, which can be observed in Figure 2. The occurrence of this phenomenon can be reduced by properly adjusting the retractions in the (PrusaSlicer, Prague, Czech Republic). The principle of retraction is to pull the filament back as the print head moves between objects, but this can affect the print quality. In this case, the filaments created did not cause problems and could be easily removed.

Two outer perimeters with 100% fill were chosen for all printed samples. For the solids with less than 100% fill density, full fill was chosen for the four lower and four upper layers (4 × 0.2 mm = 0.8 mm) due to the sample confinement.

### 2.2. Mechanical Tests

The mechanical properties of type 1B tensile test samples were investigated based on the results of tensile tests and fatigue behaviours at different intensities of progressive cyclic tensile loading for tensile test samples with different infill densities and nozzle diameters of 0.4 and 0.6 mm. For practical application, it is the testing of cyclic loading that is essential, as it provides results that are usable in practice compared to static tests. In general, it is known that cyclic tests simulate the gradual changes in mechanical properties due to different loading intensities.

Mechanical tests were performed at a laboratory temperature of 22 ± 2 °C on auniversal test machine (LABTest 5.50 ST with an AST KAF 50 kN measuring unit (LABOR-TECH s.r.o., Opava, Czech Republic) with Test and Motion software (LABORTECH s.r.o., Opava, Czech Republic), enabling cyclic tests with controlled voltage modes and load speeds. The evaluation software Test and Motion allows editing and specific settings of the fatigue test, and it also records stress and strain data during the experiment. The findings from the static tensile test results were used to determine the baseline parameters under low cyclic loading of the bamboo-filled PLA-based material, i.e., maximum load and strain.

The low-cycle loading of the test samples consisted of successive loads of 250 cycles at loads from 5 to 20, 30, 40, and 50% based on the limits found in the static tensile test (reference values used for the cyclic tests).

The cyclic loading, i.e., 1000 cycles, was divided into four consecutive cycles; the first was at 5–20% loading with 250× repetition, followed by a second loading cycle with 5–30% loading with 250× repetition, followed by a third cycle with loading in the interval of 5–40% with 250× repetition, and finally a set of cyclic loading of 5–50% with 250× repetition. A detailed description of the load variation for each cycle based on the results of the limit values found in the static tensile test can be seen in Table 2.

**Figure 3:**
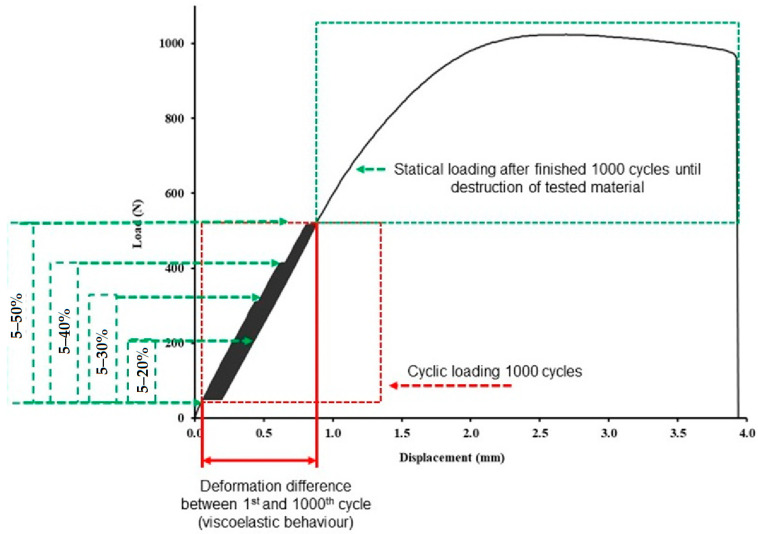
Low-cycle fatigue behaviour of 3D-printed PLA reinforced with natural bamboo reinforcement.

The tests were performed at a loading rate of 10 mm × min^−1^ between 5%, i.e., the lower limit from the established tensile static test reference value, and the upper limits of 20%, 30%, 40%, and 50%. The relaxation between every set of 1000 cycles or the endurance at the lower and upper limits was set at 0.5 s. If no destruction of the tested test sample occurred during cyclic tests, then loading was followed until the limit state was reached, i.e., destruction with the same loading rate of 10 mm × min^−1^. The cyclic fatigue tests resulted in the ultimate strength, deformation, and deformation differences between the 1st and 1000th cycles to determine the viscoelastic behaviours. Figure 3 shows the cyclic testing progress of the test samples.

### 2.3. Structural Characterization

The test samples that were destroyed during mechanical tests were subjected to image analysis of the surfaces using a MIRA 3 TESCAN GMX SE scanning electron microscope (Tescan Brno s.r.o., Brno, Czech Republic) with an accelerating voltage of 10 kV and an Oxford SE detector (Tescan Brno s.r.o., Brno, Czech Republic), while the tested surface of the test samples was gold-plated for SEM analysis with Quorum Q150R ES—Sputtering Deposition Rate (Tescan Brno s.r.o., Brno, Czech Republic).

## 3. Results and Discussion

### 3.1. Results of Mechanical Tests

The tensile strength of test samples printed by FDM technology from PLA is about 60 MPa [1]. The previously reported research results show that with the addition of different fillers to PLA, the tensile strength usually decreases. This decrease in tensile strength then increases with filler concentration [5,25,30,31]. The degree of decrease in tensile strength depends on the type, concentration, and shape of the filler. Azadi et al. reported that PLA has a better fatigue life than samples made from other polymers, e.g., ABS [18].

**Figure 4:**
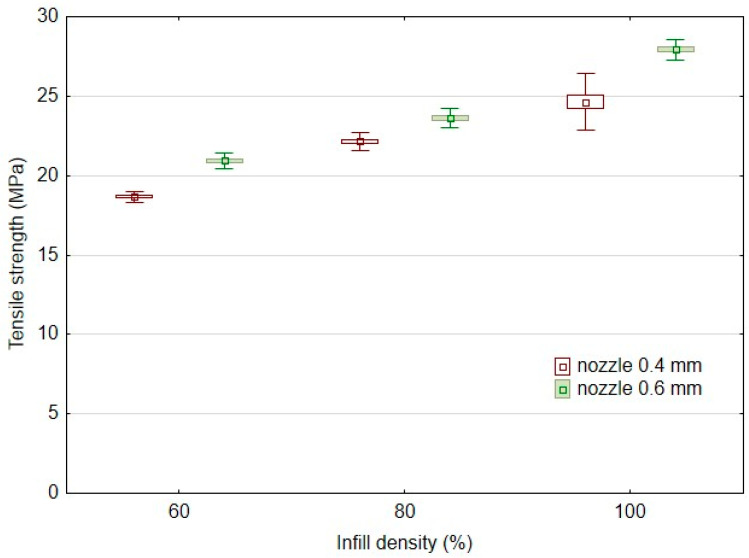
Influence of infill density on tensile strength in static testing using FDM 3D printing with different nozzle diameters.

**Figure 5:**
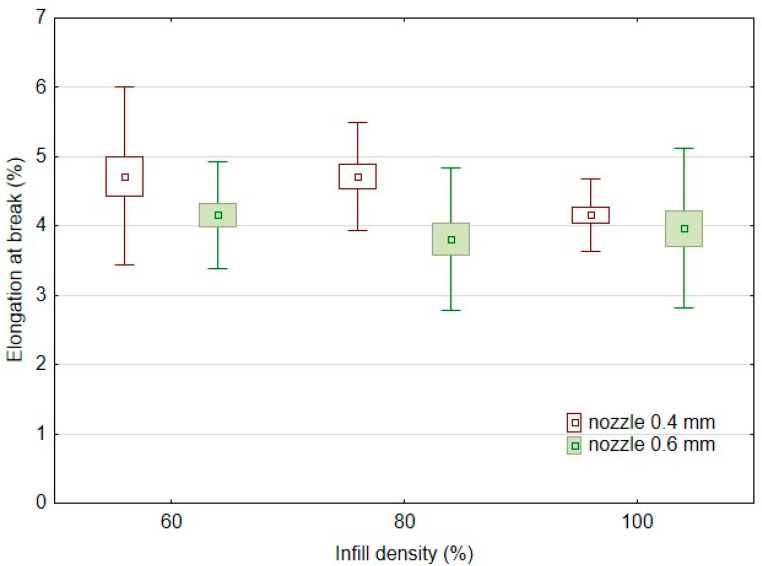
Effect of infill density on elongation at break in static testing using FDM 3D printing with different nozzle diameters.

Another significant factor affecting the mechanical behaviour of 3D-printed products is infill density. The results of the basic mechanical characteristics of the tested bamboo-filled PLA filament-based materials, i.e., tensile strength (Figure 4) and elongation at break (Figure 5), present different behaviours in static testing at different infill densities and using two different nozzles of 0.4 and 0.6 mm. The static tensile test results confirmed the significant effect of infill densities of 60%, 80%, and 100% on the tensile strength of the tested samples. Using a 0.4 mm nozzle, the static tensile strength at 100% infill density (PLA/B/nozzle 0.4 mm/100%) was 24.6 ± 0.9 MPa. The results (Figure 4) show an almost linear trend of decrease in tensile strength with decreasing infill density. For 80% infill density there was a 10% decrease in tensile strength, and for 60% infill density there was a decrease of up to 24%. A similar trend was found when using a nozzle diameter of 0.6 mm. The static tensile strength at 100% infill density (PLA/B/nozzle 0.6 mm/100%) was 27.9 ± 0.3 MPa. There was a 15% decrease in tensile strength at 80% infill density using a 0.6 mm nozzle diameter and up to a 25% decrease with 60% infill density. The arithmetic mean of the tensile strength results also shows that there was a 6–12% increase in tensile strength when using a larger nozzle diameter, i.e., 0.6 mm.

It is evident from the research on adding fillers to PLA filaments that apart from a reduction in strength, there is usually also an increase in elongation at break [25,30,31]. The elongation at break for the tested samples printed by FDM from PLA was about 3.5% [1].

Figure 5 presents the elongation at break for static testing at different infill densities and using two different nozzles of 0.4 and 0.6 mm. From the results, it is clear that there is no significant change due to different nozzle diameters and infill densities of 60, 80, and 100% when considering the variance of the results plotted in Figure 5. It is also evident from the results of the arithmetic mean of elongation at break that there is no significant change when using a larger nozzle diameter. When a nozzle diameter of 0.4 mm was used, the elongation at break was 4.16 ± 0.26% at 100% infill density for the sample labelled PLA/B/nozzle 0.4 mm/100%. At 80% and 60% infill densities, there was an identical 13% increase in elongation at break. Using a 0.6 mm diameter nozzle, elongation at break was 3.97 ± 0.87% at 100% infill density for the sample labelled PLA/B/nozzle 0.6 mm/100. At 80% infill density, using a 0.6 mm diameter nozzle, there was a 4% decrease in elongation at break, but at 60% infill density, there was a 4.8% increase in elongation at break.

**Figure 6:**
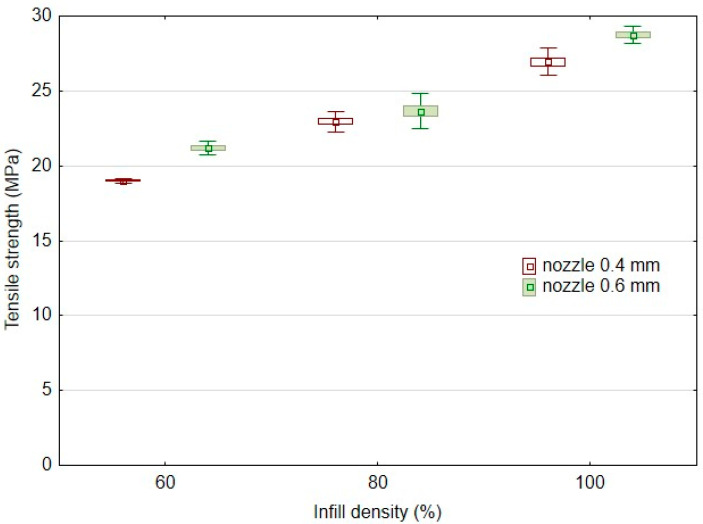
Effect of infill density on tensile strength in low-cycle testing using FDM 3D printing with different nozzle diameters.

The results of the tensile strength after the low-cycle test can be seen in Figure 6, which consisted of 1000 cycles divided into four consecutive load intervals from 5 to 20, 30, 40, and 50% based on the limit values found in the static tensile test divided into 250 consecutive cycles. The research results showed minimal effects on the low-cycle tensile properties according to the conditions defined in Table 2 using additive manufacturing by 3D printing using the FDM method from PLA-based filament and bamboo filler. All test samples withstood successive cyclic loading at different loading force intensities (Table 3). The results are consistent with previous findings dealing with fatigue life and showed a positive or neutral effect of the filler. For all experimental variations, low-cycle testing resulted in an increase in tensile strength over the static tensile test values ranging from 0.2 to 9.4% [10,28]. This trend is demonstrated by Senatov et al. in their research, i.e., there is a cyclic strengthening of the material [29]. The higher strengthening after low-cycle testing relative to the static test values occurred for 3D printing with a nozzle of 0.4 mm, i.e., in the interval from 1.8 to 9.4%. Another finding is that the increase of 2.9 to 9.4% in tensile strength after low-cycle testing occurred for materials with an infill density of 100%. As the infill density decreased, the final tensile strength after low-cycle test decreased. From the point of view of practical application, the finding that low-cycle fatigue does not reduce the tensile strength up to 50% of the load is significant.

The results of the static tensile test after the low-cycle test also confirmed the significant effect of the infill densities of 60%, 80%, and 100% on the tensile strengths of the tested samples. The results (Figure 6) show an almost linear trend of decreases in tensile strength after low-cycle testing with decreasing infill densities. Using a 0.4 mm diameter nozzle, the tensile strength after low-cycle testing for 100% infill density (PLA/B/nozzle 0.4 mm/100%) was 27.0 ± 0.5 MPa. At 80% infill density, the tensile strength after the low-cycle test decreased by 15%, and at 60% infill density, it decreased by up to 29.6%. A similar trend was found when using a 0.6 mm diameter nozzle. The tensile strength after low-cycle testing at 100% infill density (PLA/B/nozzle 0.6 mm/100%) was 28.7 ± 0.3 MPa. At 80% infill density there was a decrease in tensile strength after low-cycle testing by 17.6%, and at 60% infill density, it decreased by up to 26.2%. The results presented by Essassi et al. also showed the importance of density on fatigue properties [30].

**Figure 7:**
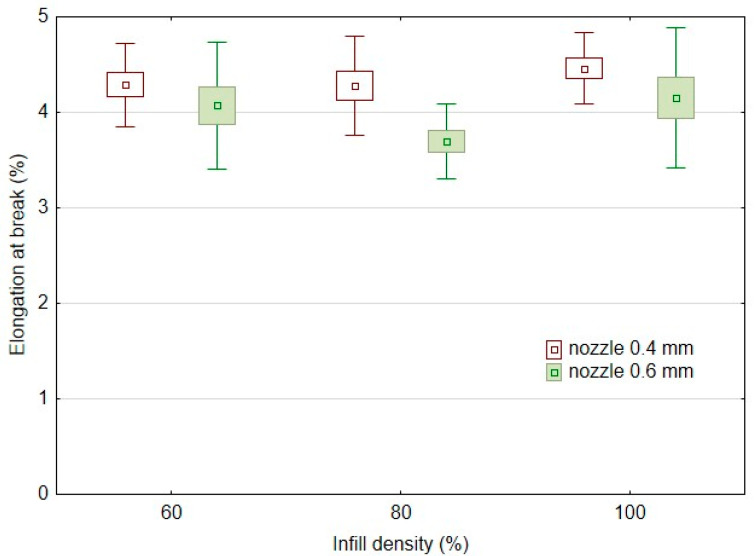
Effect of infill density on elongation at break in low-cycle testing using FDM 3D printing with different nozzle diameters.

From the results of the elongation at break after low-cycle testing observed in Figure 7, it is clear that there is no significant change due to the change of the nozzle diameter and the infill densities of 60%, 80%, and 100% considering the variance of the results. The results of the arithmetic mean of the elongation at break after low-cycle test show a similar trend to that found in the static tensile test (Figure 5). When a larger nozzle diameter, i.e., 0.6 mm, was used, the elongation at break after low-cycle testing was reduced by 5 to 16%. When a nozzle diameter of 0.4 mm was used, the elongation at break after low-cycle testing was 4.46 ± 0.19% at 100% infill density for the sample labelled PLA/B/nozzle 0.4 mm/100%. At 80% and 60% infill densities, there was an increase in elongation at break after low-cycle testing of 3.8 to 4.1%. Using a 0.6 mm diameter nozzle, the elongation at break after low-cycle testing was 4.15 ± 0.37% for the 100% infill density sample labelled PLA/B/nozzle 0.6 mm/100. At 80% infill density using a 0.6 mm nozzle, there was an 11% decrease in elongation at break after the low-cycle testing, and at 60% infill density there was also a 2.0% decrease.

The low-cycle testing resulted in an increase in elongation at break compared to the static tensile test values only for 100% infill density, ranging from 4.6 to 7.1%. For the other experimental variants having 80 and 60% infill densities, the low-cycle test resulted in a decrease in elongation at break relative to the values found in the static test. These results showed a decrease in deformation under low-cycle testing. When using a 0.4 mm nozzle the deformation was decreased from 9.1 to 9.3%. When using a 0.6 mm nozzle the deformation decreased from 2.1 to 3.0%.

**Figure 8:**
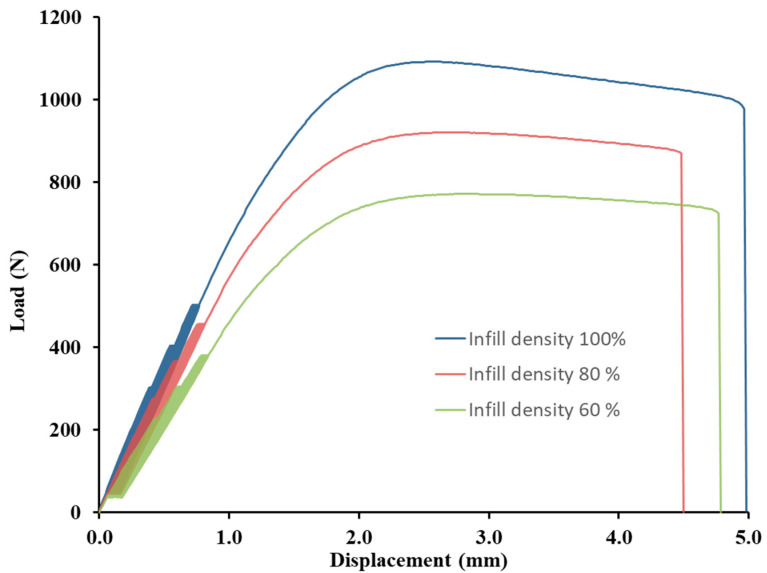
Influence of infill density on low-cycle fatigue using FDM 3D printing with a 0.4 mm nozzle diameter.

**Figure 9:**
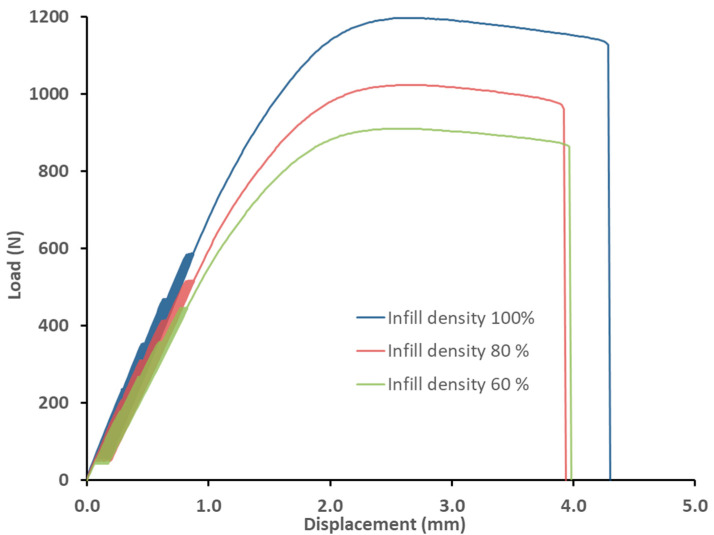
Influence of infill density on low-cycle fatigue using FDM 3D printing with a 0.6 mm nozzle diameter.

Table 3 shows the difference in deformation between the first and the last (i.e., 1000) test cycles for each tested variant. This difference between the first and last test cycles represents the shift of the hysteresis loops for the low-cycle tests (Figure 8 and Figure 9) and represents the viscoelastic behaviour or creep of the tested experimental variants. Examples of the loading curves and low-cycle fatigue test progress are shown in Figure 8 for the 0.4 mm diameter nozzle and in Figure 9 for the 0.6 mm diameter nozzle. The results show the deformation between the first and last test cycles (1000) of the test specimens during the low-cycle test, which presents the fatigue behaviour of the printed test samples by FDM 3D printing technology of bamboo-filled PLA material at different infill densities and using two nozzle diameters. The difference between the 1st and 1000th cycle ranged from 0.09 to 0.11%. From the results shown in Table 3, there is no clear trend of the difference between the 1st and 1000th cycle depending on the nozzle diameter and infill density. It can only be stated that the 0.6 mm nozzle showed similar values of difference between the 1st and 1000th cycle at different infill densities. However, it is clear from the results that the low-cycle test showed a creep of the test samples during loading. This creep was manifested by a deformation characterized by the difference in strain between the first and last cycle. This trend is also evident from the shift of the hysteresis loops shown in Figure 8 and Figure 9. After stress relief, the hysteresis curve should tend to return to its original state. This would prevent material creep. The accumulated stress during low-cycle fatigue induces deformation that leads to different magnitudes of hysteresis loop displacement, which can be seen from Figure 8 and Figure 9 and also from the results shown in Table 3, i.e., the difference in deformation between cycles 1 and 1000.

If this strain exceeded the elastic limit, significant plastic deformation would occur, leading to premature destruction of the test samples [1]. However, this did not happen, and all test samples withstood the specified 1000 load cycles. Tao and Xia stated that the rate and progression of loading will have a significant effect on the fatigue behaviours of polymeric materials. Therefore, it is very important to obtain strain and stress data during the fatigue process [32].

### 3.2. SEM Analysis of Tested Materials

**Figure 10:**
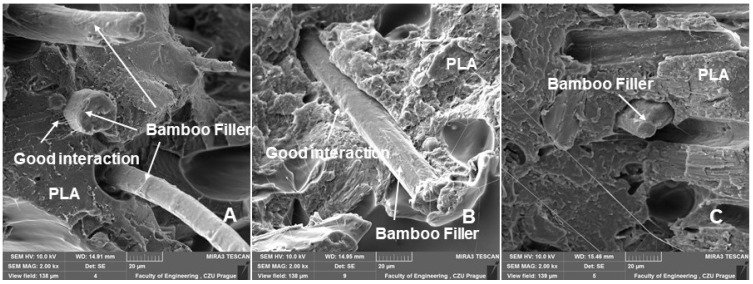
SEM images—fractographic analysis (MAG 2.00 kx): (**A**): nozzle 0.4 mm, infill density 60%, static test; (**B**): nozzle 0.4 mm, infill density 80%, static test; (**C**): nozzle 0.6 mm, infill density 100%, low cyclic fatigue.

**Figure 11:**
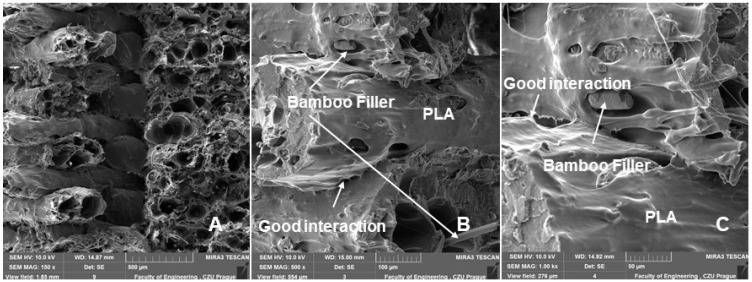
SEM images—fractographic analysis—nozzle 0.6 mm, infill density 60%, static tensile test—connection of individual layers, arrangements, and their porosities: (**A**): MAG 150×; (**B**): MAG 500×; (**C**): MAG 1.00 kx.

**Figure 12:**
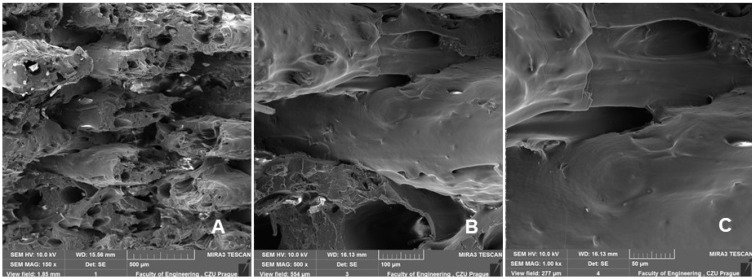
SEM images—fractographic analysis—nozzle 0.4 mm, infill density 60%, low cyclic fatigue—connection of individual layers, arrangements, and their porosities: (**A**): MAG 150×; (**B**): MAG 500×; (**C**): MAG 1.00 kx.

**Figure 13:**
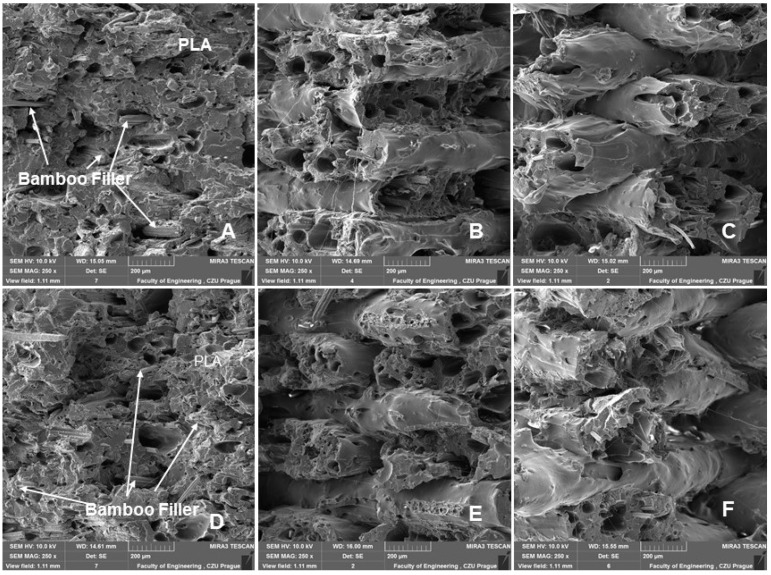
SEM images—fractographic analysis—nozzle 0.4 mm (MAG 2.50×): (**A**): Infill density 100%, static tensile test; (**B**): Infill density 80%, static tensile test; (**C**): Infill density 60%, static tensile test; (**D**): Infill density 100%, low cyclic fatigue; (**E**): Infill density 80%, low cyclic fatigue; (**F**): Infill density 60%, low cyclic fatigue.

**Figure 14:**
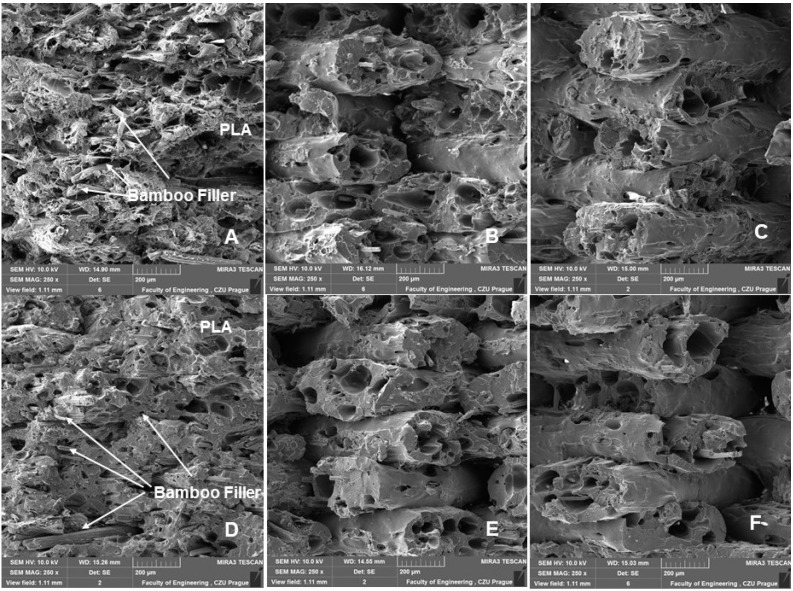
SEM images—fractographic analysis—nozzle 0.6 mm (MAG 2.50×): (**A**): Infill density 100%, static tensile test; (**B**): Infill density 80%, static tensile test; (**C**): Infill density 60%, static tensile test; (**D**): Infill density 100%, low cyclic fatigue; (**E**): Infill density 80%, low cyclic fatigue; (**F**): Infill density 60%, low cyclic fatigue.

SEM images of the fracture surface using 3D printing with FDM of a bamboo-filled PLA-based material with infill densities and nozzle diameters of 0.4 and 0.6 mm can be seen in Figure 10, Figure 11, Figure 12, Figure 13 and Figure 14. These images present the interactions of the bamboo fillers and PLA matrices, the structures and sizes of the layers affected by infill density and nozzle diameter, and finally the changes caused by low-cycle fatigue. From the fracture surface shown in Figure 10, a good interaction of the bamboo filler with the matrix can be seen when using nozzle diameters of 0.4 and 0.6 mm. The results are consistent with the claim that the biofiller has a good interfacial interaction with the PLA matrix [27]. This is the first prerequisite for the successful use of the tested bamboo-filled PLA-based filament. During 3D printing by the FDM method, rapid diffusion occurs during high-temperature processing, which causes interdiffusion bonding between the biofiller and PLA, which may result in good contact between the matrix and the filler and could improve the resulting mechanical properties [10].

Figure 11 and Figure 12 are examples of figures showing the connection of individual layers, their arrangement, and their porosity. Figure 12 and Figure 13 show consistently good bonding of the individual layers. The assumption of Chen et al. that poor interlayer bonding occurs when using FDM technology, leading to anisotropy in mechanical properties, is not confirmed [33]. Figure 11 and Figure 12A,B show the porosity that usually occurs in nonideal manufacturing during 3D printing and in materials with natural fillers [30,34]. The weight of the test sample fabricated by 3D printing from PLA was 9.95 g. For PLA with bamboo-based filler, the weight ranged from 7.40 g to 8.91 g. The weight varied due to infill density. There was an increase in weight with increasing infill density value. At 100% infill density, the weight was found to be 8.87 g using a 0.4 mm diameter nozzle and 8.91 g using a 0.6 mm diameter nozzle.

Figure 13 and Figure 14 show a comparison of 3D printing with nozzle diameters of 0.4 and 0.6 mm. Figure 13 and Figure 14A–C present the SEM results of the fractographic analysis after tensile testing. Figure 13 and Figure 14D–F present the results of SEM fractographic analysis after low-cycle fatigue. From Figure 13 and Figure 14, the effect of infill densities of 100%, 80%, and 60% can be compared. In their research, Jerez-Mesa et al. found that the layer height had a significant effect on the fatigue life in PLA [35]. For this reason, the fracture surface seen in the SEM images was subjected to image analysis using Gwyddion software, the results of which can be observed in Table 4.

Jerez-Mesa et al. reported that with increasing height of the PLA layer of the test samples, better results were found in the number of cycles for resisting failure [35]. This was not demonstrated in the research. The test samples withstood the entire low-cycle fatigue test cycle. From the results shown in Table 4, the trend of the difference in the layer found from SEM image fractographic analysis during static and low-cycle fatigue testing is not clear. However, the effect of infill density is evident from the results. There was a decrease in layer height with an increasing infill density value (Table 4).

There was no reduction in sample height, pore collapse, or delamination due to cyclic loading, as indicated in the research of Senatov et al. [29].

## 4. Conclusions

In this article, results focused on 3D printing using PLA-based filament with natural bamboo filler are reported. The development of materials with natural filler is very important in the field of additive technologies. Bamboo-based natural filler contributes significantly to the improvement of aesthetic properties. The results demonstrated the suitability of bamboo-filled PLA filament for 3D printing applications, with emphasis on fatigue behaviour under different intensities of successive load cycles. The obtained results of controlled loading during cyclic tests demonstrated the functionality of the tested material at the evaluated manufacturing parameters affecting the durability of potential 3D printed products made from PLA-based filament filled with bamboo filler subjected to low-cycle fatigue. Cyclic stresses represent a common cause of failure of their adhesive bonds, thereby reducing their service life.

Based on the results presented in this article, the following conclusions can be drawn:

- The static tensile test results confirmed the significant effects of infill densities of 60%, 80%, and 100% on the tensile strength of the tested samples and of 0.4 and 0.6 mm printing nozzles on the tensile strength. In particular, the research results show the significant effect of infill density on the fatigue properties of the tested materials.
- For all tested variants of the experiment, the low-cycle test resulted in an increase in tensile strength compared to the values from the static test, up to about 10%. Thus, a so-called cyclic strengthening of the material occurred. The viscoelastic behaviour (creep) of the material during low-cycle fatigue was also evident. Infill density again proved to have a significant influence. At 100% infill density, the highest fatigue tensile strength was achieved.
- SEM analysis of the fracture surface confirmed good interaction between the PLA matrix and the bamboo-based filler using the tested print nozzle diameters of both 0.4 and 0.6 mm.

The results of SEM analysis did not show any reductions in the layer height of the samples, pore collapse, or delamination due to low cyclic loading. However, they showed the effect of infill density on the layer height of the samples.

## Figures and Tables

**Table 1:** Parameters for the printing samples.

Printing Variant	Nozzle ø (mm)	Infill Density (%)	Printing Time (min)	Amount of Filament (m)
PLA/B/nozzle 0.4 mm/60% *	0.4	60	35	2.87
PLA/B/nozzle 0.4 mm/80%	0.4	80	38	3.15
PLA/B/nozzle 0.4 mm/100%	0.4	100	39	3.43
PLA/B/nozzle 0.6 mm/60%	0.6	60	25	2.97
PLA/B/nozzle 0.6 mm/80%	0.6	80	27	3.23
PLA/B/nozzle 0.6 mm/100%	0.6	100	27	3.48

* PLA—polylactic acid/B—bamboo filler/nozzle 0.4 mm—used nozzle/60%—infill density.

**Table 2:** Low-cycle test setup parameters for each experiment variant tested.

Printing Variant	Description of the Low-Cycle Stresses for the Individual Printed Test Variants
PLA/B/nozzle 0.4 mm/60%	The low-cycle loading was from 5% (45 N) to 20% (152 N) with a repetition of 250 cycles, followed by a second loading cycle from 5% to 30% (228 N) 250 cycles, followed by a third loading cycle from 5% to 40% (304 N) 250 cycles, and a final loading cycle from 5% to 50% (379 N) 250 cycles.
PLA/B/nozzle 0.4 mm/80%	The low-cycle loading was from 5% (45 N) to 20% (181 N) with a repetition of 250 cycles, followed by a second loading cycle from 5% to 30% (271 N) 250 cycles, followed by a third loading cycle from 5% to 40% (362 N) 250 cycles, and a final loading cycle from 5% to 50% (452 N) 250 cycles.
PLA/B/nozzle 0.4 mm/100%	The low-cycle loading was from 5% (50 N) to 20% (199 N) with a repetition of 250 cycles, followed by a second loading cycle from 5% to 30% (299 N) 250 cycles, followed by a third loading cycle from 5% to 40% (399 N) 250 cycles, and a final loading cycle from 5% to 50% (499 N) 250 cycles.
PLA/B/nozzle 0.6 mm/60%	The low-cycle loading was from 5% (44 N) to 20% (178 N) with a repetition of 250 cycles, followed by a second loading cycle from 5% to 30% (267 N) 250 cycles, followed by a third loading cycle from 5% to 40% (356 N) 250 cycles, and a final loading cycle from 5% to 50% (445 N) 250 cycles.
PLA/B/nozzle 0.6 mm/80%	The low-cycle loading was from 5% (52 N) to 20% (206 N) with a repetition of 250 cycles, followed by a second loading cycle from 5% to 30% (309 N) 250 cycles, followed by a third loading cycle from 5% to 40% (412 N) 250 cycles, and a final loading cycle from 5% to 50% (515 N) 250 cycles.
PLA/B/nozzle 0.6 mm/100%	The low-cycle loading was from 5% (58 N) to 20% (232 N) with a repetition of 250 cycles, followed by a second loading cycle from 5% to 30% (348 N) 250 cycles, followed by a third loading cycle from 5% to 40% (464 N) 250 cycles, and a final loading cycle from 5% to 50% (580 N) 250 cycles.

**Table 3:** Results of deformation of the test samples between the first and last test cycles in the low-cycle test.

Printing Variant	Number of Finished Tests	Relative Deformation 1st Cycle (%)	Relative Deformation 1000th Cycle (%)	Strain Difference between 1st and 1000th Cycle (%)
PLA/B/nozzle 0.4 mm/60%	1000	0.069 ± 0.005	0.174 ± 0.005	0.11 ± 0.01
PLA/B/nozzle 0.4 mm/80%	1000	0.072 ± 0.005	0.165 ± 0.014	0.09 ± 0.01
PLA/B/nozzle 0.4 mm/100%	1000	0.065 ± 0.000	0.159 ± 0.016	0.09 ± 0.02
PLA/B/nozzle 0.6 mm/60%	1000	0.069 ± 0.005	0.171 ± 0.011	0.11 ± 0.01
PLA/B/nozzle 0.6 mm/80%	1000	0.072 ± 0.005	0.184 ± 0.005	0.11 ± 0.00
PLA/B/nozzle 0.6 mm/100%	1000	0.078 ± 0.005	0.184 ± 0.005	0.10 ± 0.01

**Table 4:** Results of layer height measurements determined from SEM image analysis.

Printing Variant	Layer Height—Static Test (mm)	Layer Height- Low-Cycle Fatigue (mm)
PLA/B/nozzle 0.4 mm/60%	0.22 ± 0.02	0.23 ± 0.02
PLA/B/nozzle 0.4 mm/80%	0.20 ± 0.02	0.22 ± 0.32
PLA/B/nozzle 0.4 mm/100%	0.18 ± 0.03	0.18 ± 0.02
PLA/B/nozzle 0.6 mm/60%	0.21 ± 0.02	0.21 ± 0.03
PLA/B/nozzle 0.6 mm/80%	0.20 ± 0.02	0.20 ± 0.01
PLA/B/nozzle 0.6 mm/100%	0.18 ± 0.02	0.19 ± 0.02

## Data Availability

Not applicable.
